# Supplementary material for: Risk factors for sacrococcygeal pilonidal sinus: a systematic review and meta-analysis supplemented by genetic causal assessment
Source: Front Surg. 2026 Jan 7;12:1718589. doi: 10.3389/fsurg.2025.1718589 (PMC12819706; doi:10.3389/fsurg.2025.1718589)
Supplement: Supplementary file 2 [file Datasheet2.zip › Supplementary Data 2/MR_pipeline_p5e-6/leaveone_plots/finngen_R12_L12_HIDRADENITISSUP.finngen_R12_L12_PILONIDALCYST.pdf]

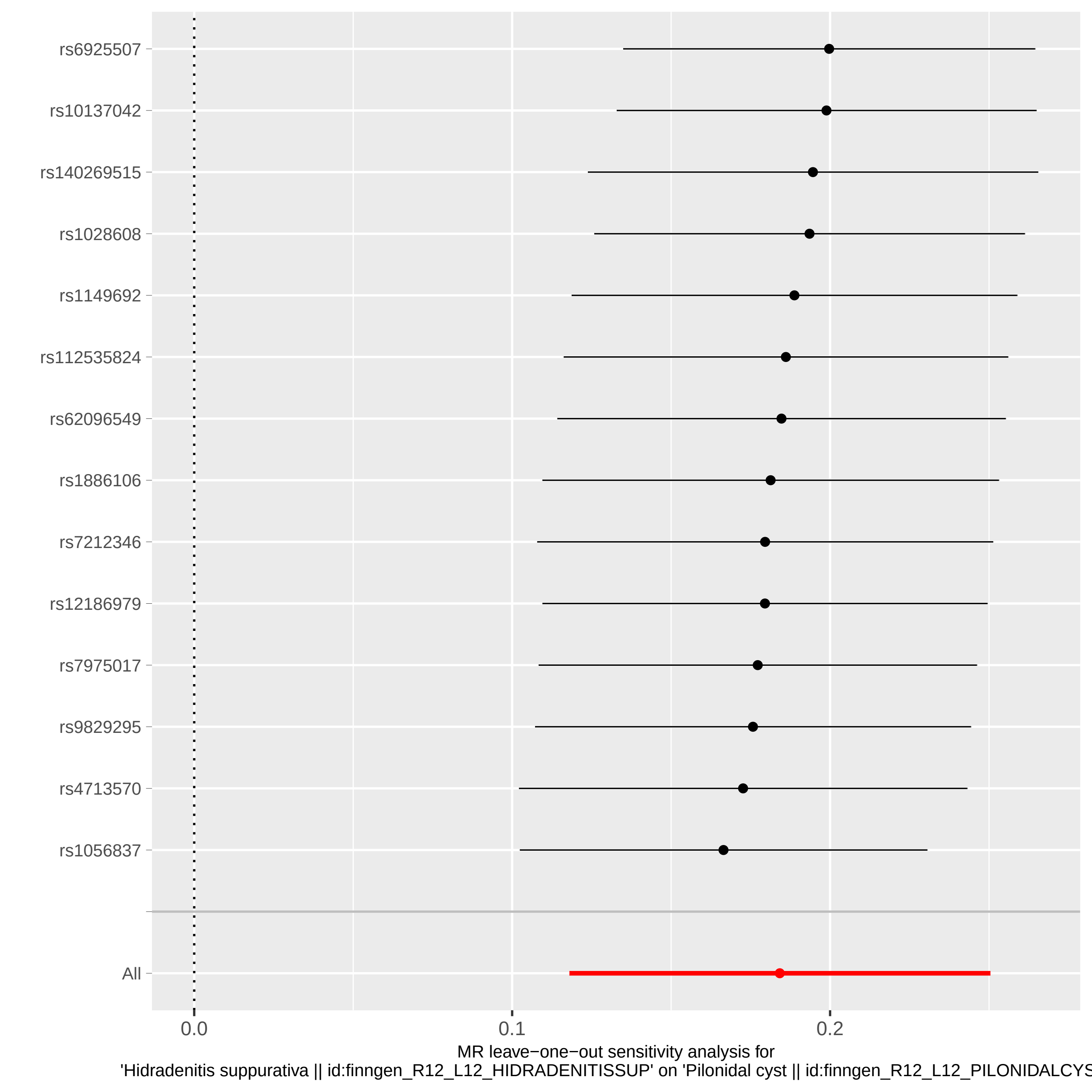
rs6925507
rs10137042
rs140269515
rs1028608
rs1149692
rs112535824
rs62096549
rs1886106
rs7212346
rs12186979
rs7975017
rs9829295
rs4713570
rs1056837
All
0.0
0.1
0.2
MR leave−one−out sensitivity analysis for
'Hidradenitis suppurativa || id:finngen_R12_L12_HIDRADENITISSUP' on 'Pilonidal cyst || id:finngen_R12_L12_PILONIDALCYS
